# Retraction Note: Biological interactions between micro swimmers and cross fluid with inclined MHD effects in a complex wavy canal

**DOI:** 10.1038/s41598-024-66666-x

**Published:** 2024-07-10

**Authors:** Assad Ayub, Syed Zahir Hussain Shah, Muahmmad Imran Asjad, Musawa Yahya Almusawa, Sayed M. Eldin, Magda Abd El-Rahman

**Affiliations:** 1 Department of Mathematics and Statistics, Hazara University, Manshera, Pakistan; 2 Department of Mathematics, Government College Mansehra, Mansehra, 21300 Pakistan; 3 Department of Mathematics, University of Management and Technology, Lahore, Pakistan; 4 Department of Mathematics, Faculty of Science, Jazan University, 45142 Jazan, Saudi Arabia; 5 Center of Research, Faculty of Engineering and Technology, Future University in Egypt, New Cairo, 11835 Egypt; 6 Department of Physics, College of Science, King Khalid University, 61413 Abha, Saudi Arabia; 7 Department of Radiation Physics, National Centre of Radiation Research and Technology (NCRRT), Atomic Energy Authority, Cairo, 11787 Egypt

Retraction of: *Scientific Reports* 10.1038/s41598-023-31853-9, published online 22 March 2023

The Editors have retracted this Article.

After publication of this Article, concerns about similarities between this Article and a previous article with no common authors^1^ were reported to the Editors. Figures 13–16 each appear to have been previously published in the earlier paper^1^, and Figures 10–12 each report data that is very similar to data previously published in the earlier paper. Further review by the Editors also identified additional concerns regarding Figures 3–5, which each display three curves for four values of the parameter omega, and the insets of Figures 4 and 7, where the values of the labels are discrepant with the values of the main axes. The Editors no longer have confidence in the reliability of the findings and conclusions of this Article.

Assad Ayub, Syed Zahir Hussain Shah, Muahmmad Imran Asjad, and Musawa Yahya Almusawa disagree with the retraction. Magda Abd El-Rahman did not respond to the correspondence from the Editors about the retraction. Editors were not able to confirm the current contact details for Sayed M. Eldin.
